# Cardiovascular effects of cholecalciferol treatment in dialysis patients – a randomized controlled trial

**DOI:** 10.1186/1471-2369-15-50

**Published:** 2014-03-24

**Authors:** Frank H Mose, Henrik Vase, Thomas Larsen, Anne SP Kancir, Renata Kosierkiewic, Bartlomiej Jonczy, Annebirthe B Hansen, Anna E Oczachowska-Kulik, Ingrid M Thomsen, Jesper N Bech, Erling B Pedersen

**Affiliations:** 1 University Clinic in Nephrology and Hypertension, Department of Medical Research and University of Aarhus, Holstebro Hospital, Holstebro, Denmark; 2 Department of Medicine, Holstebro Hospital, Laegaardvej 12, Holstebro 7500, Denmark; 3 Department of Clinical Biochemistry, Holstebro Hospital, Holstebro, Denmark; 4 Department of Cardiology, Aarhus University Hospital, Aarhus, Denmark

**Keywords:** Cholecalciferol, Chronic kidney disease, Cardiac function, Brain natriuretic peptide, Blood pressure

## Abstract

**Background:**

Patients on chronic dialysis are at increased risk of vitamin D deficiency. In observational studies plasma 25-hydroxyvitamin D (p-25(OH) D) levels are inversely correlated with plasma BNP and adverse cardiovascular outcomes. Whether a causal relation exists has yet to be established. The aim of this study was to test the hypothesis that cholecalciferol supplementation improves cardiac function and reduces blood pressure (BP) and pulse wave velocity (PWV) in patients on chronic dialysis.

**Methods:**

In a randomized, placebo-controlled, double-blind study, we investigated the effect of 75 μg (3000 IU) cholecalciferol daily for 6 months, in patients on chronic dialysis. We performed two-dimensional echocardiography, with doppler and tissue-doppler imaging, 24-h ambulatory BP (24-h BP), PWV, augmentation index (AIx), central BP (cBP) and brain natriuretic peptide (BNP) measurements at baseline and after 6 months.

**Results:**

Sixty-four patients were allocated to the study. Fifty dialysis patients with a mean age of 68 years (range: 46–88) and baseline p-25(OH) D of 28 (20;53) nmol/l completed the trial. Cholecalciferol increased left ventricular (LV) volume, but had no impact on other parameters regarding LV structure or left atrial structure. LV systolic function, LV diastolic function, PWV, cBP, AIx and BNP were not changed in placebo or cholecalciferol group at follow-up. 24-h BP decreased significantly in placebo group and tended to decrease in cholecalciferol group without any difference between treatments.

**Conclusion:**

Six months of cholecalciferol treatment in patients on chronic dialysis did not improve 24-h BP, arterial stiffness or cardiac function.

**Trial registration:**

NCT01312714, Registration Date: March 9, 2011.

## Background

Patients with CKD have profound reductions in plasma levels of both 25-hydroxyvitamin D [25(OH) D] and the more potent 1,25 dihydroxyvitamin D [1,25(OH)_2_D] [1-4]. In patients with CKD, low 25(OH) D levels are associated with higher brain natriuretic peptide (BNP) levels [5], increased cardiovascular events [6,7] and increased mortality [8]. However, these observations are prone to confounding, because healthier people may spend more time outdoors.

The importance of vitamin D to mineral metabolism is established but vitamin D may possess beneficial effects on the cardiovascular system in patients with CKD. The vitamin D receptors are widely expressed in human tissues, and recent studies have also found active 1α-hydroxylase in extra-renal tissues, in including cardiac tissue [9] and vascular smooth muscle cells [10,11]. In addition, extra-renal produced 1,25(OH)_2_D seems to have important paracrine and autocrine effects [12,13], which may be highly relevant in patients with reduced renal mass and impaired renal 1,25(OH)_2_D production.

Active vitamin D analogue treatment is associated with improved survival in dialysis patients [14], and in spontaneously hypertensive rats active Vitamin D analogues prevented left ventricular hypertrophy [15]. In dialysis patients plasma brain natriuretic peptide (p-BNP) is a predictor of all-cause mortality [16] and cholecalciferol supplementation was associated with reduced p-BNP and reduced left ventricular mass (LVM) [3,17].

Randomized controlled trials establishing the effect of cholecalciferol supplementation on cardiac function and BP in patients with CKD are still mandated. The aim of this study was to test the hypothesis that cholecalciferol supplementation improves cardiac function and reduces BNP, 24 hour ambulatory BP (24-h BP) and pulse wave velocity (PWV) in patients with stage 5 CKD on chronic dialysis.

## Methods

### Study population

Subjects passed a screening examination that included medical history and physical examination. *Inclusion criteria*: Both men and women, age above 18 years, dialysis treatment for more than 3 months. *Exclusion criteria*: Current malignant disease, hypercalcemia (albumin corrected serum calcium > 2.60 mmol/L), allergy or intolerance towards cholecalciferol tablets, incapability to give informed consent. *Withdrawal criteria*: Renal transplantation, non-compliance, clinical complications rendering oral ingestion of tablets or trial completion impossible, transferral to another dialysis department and withdrawal of informed consent.

### Design

The study was a single-center, randomized, placebo-controlled, double blinded, trial. After inclusion subjects were allocated to treatment via computer-generated randomization in blocks of ten and received 3000 IU (75 μg) cholecalciferol daily or placebo for 6 months.

### Study drugs

Cholecalciferol and placebo were identical in appearance and were given orally and were obtained from Ferrosan A/S, Soeborg, Denmark.

### Ethics

The study was approved by the Regional Committee on Biomedical Research Ethics, and carried out in accordance with the Declaration of Helsinki. Written, informed consent was obtained from each subject.

### Effect variables

The main effect variable was p-BNP. Other effect variables were 24-h BP, central diastolic BP (cDBP), central systolic BP (cSBP), PWV, AIx and plasma concentrations of renin (PRC), angiontensin II (p-AngII), aldosterone (p-Aldo), 25(OH) D, intact parathyroid hormone (p-PTH), ionized calcium (p-Ca^2+^), phosphate (p-phosphate), alkaline phosphatase and C-reactive protein (p-CRP). In echocardiography, structural parameters included left ventricular mass index (LVMI), Left ventricular (LV) wall thickness and LV diastolic volume and diameter and functional parameters. Functional parameters included LV Ejection Fraction, LV longitudinal function assessed by tissue Doppler, LV global and local strain assessed by 2D Speckle Tracking, and LV diastolic function assessed by early mitral inflow wave velocity/early diastolic mitral annular velocity ratio (E/e’), early/late mitral inflow wave velocity ratio (E/A) and E-wave deceleration time.

### Recruitment

Patients were consecutively recruited from the Dialysis Department and Outpatients’ Clinic of Renal Diseases, Holstebro Hospital All patients in our dialysis department were screened for participation.

### Number of subjects

With a significance level of 5% and a power of 80% a total of 56 subjects (28 in each group) were needed to detect a 15 pmol/l difference in p-BNP (SD 20 pmol/l). The aim was to include 80 patients due to an expected withdrawal of 25%. The power calculation was based on previous observational data of cholecalciferol supplementation in hemodialysis patients) [3].

### Experimental procedure

Vitamin D supplementation of more than 10 μg of ergo- or cholecalciferol daily was paused 3 months prior to allocation treatment group and baseline measurements. Transthoracic echocardiography (TTE), applanation tonometry and 24-h BP was performed at baseline and repeated after 6 months of treatment. Measurements were performed on the same day. After arrival at the research facility, TTE was performed followed by applanation tonometry and venous blood sampling for evaluation of vasoactive hormones (PRC, p-Ang II, p-Aldo, p-AVP and p-BNP). In peritoneal dialysis (PD) patients 24-h BP equipment was mounted after blood sampling. In hemodialysis (HD) patients a regular dialysis was performed after TTE, applanation tonometry and blood sampling, and 24-h BP equipment was mounted after the dialysis session had finished. In HD patients, baseline and follow-up measurements were performed on a dialysis day, with an interdialytic interval as short as possible prior to the measurements. Measurements in PD patients were performed in relation to a visit at the outpatient clinic. At the day of baseline and follow-up examinations participants took their usual medication, and were not allowed any alcoholic or caffeinated beverages. At baseline and every 4 weeks blood samples were obtained for assessment of plasma concentrations Ca^2+^, phosphate, PTH and p-25(OH) D.

### BP measurements

24-h BP was measured using Kivex TM-2430 (Kivex, Hoersholm, Denmark). Equipment was mounted after cessation of a dialysis session. Measurements were taken every 15 minute during daytime and every 30 minute over night. Brachial blood pressure used for calibration of Sphygmocor system was measure as duplicate recording with Omron 705IT (Omron Matsusaka CO. Ltd., Matsusaka City, Japan). If measurements of the duplicate recording exceeded 7 mmHg, the BP measurement was repeated.

### TTE measurements

TTE was performed using a Vivid 7 (GE Medical Systems Inc., Horten, Norway) cardiac ultrasound system. All images were stored for offline analysis. All measures was performed separately, randomly, and blinded to treatment allocation.

Left ventricular (LV) dimensions were obtained from the parasternal long-axis view, with measurement of end-diastolic interventricular septal thickness, LV posterior wall thickness, and LV end-diastolic diameter just below the tips of the anterior mitral leaflet. LV mass was calculated using the Devereux formula and indexed to body surface area to obtain the LV mass index (LVMI) [18]. LV ejection fraction (LVEF) and LV volumes were calculated from conventional apical two- and four-chamber images, using the biplane Simpson technique.

Left atrial (LA) volume was assessed by the biplane area-length method from apical 4- and 2-chamber views at end systole from the frame preceding mitral valve opening. Left atrial volume index was calculated as LA volume to body surface area (mL/m2). Mitral inflow was assessed in the apical 4-chamber view with the pulsed wave Doppler sample volume placed at the tips of mitral leaflets during diastole. From the inflow, peak E wave velocity, E wave deceleration time and peak A wave were measured. Mitral annular motion was assessed using pulsed wave tissue Doppler with the sample volume placed in the septal and lateral mitral annulus. The mean of the septal and lateral e’ velocity was used for calculation of E/e’. Global and regional strain was measured by 2D speckle tracking. All measurements were estimated as averages of three (sinus rhythm) or five (atrial fibrillation) consecutive heart beats.

### Applanation tonometry

After echocardiography, subjects were transferred to the dialysis department and supine position was assumed in a quiet temperature controlled room (temperature range 21–24°C). After 30 minutes supine position, applanation tonometry was performed. Recordings of pulse wave analysis (PWA) and carotid-femoral PWV were obtained by applanation tonometry (SphygmoCor® CPV system®, AtCor Medical, Sydney, Australia) as double-recordings by a trained observer. Only duplicate recording meeting the quality requirements were included in the final analysis [19].

### Biochemical analyses

Routine blood samples obtained at baseline and every month through out the study were immediately assayed at the Department of Clinical Biochemistry. Commercial chemiluminescence immunoassays were used to analyze plasma concentrations of 25(OH) D2 + 25(OH) D3 (Liaison, DiaSorin, Saluggia, Italy) and BNP (Centaur, Bayer Germany).

Blood samples for measurements of vasoactive hormones were centrifuged for 10 minutes at 2200 G and 4°C. Plasma was separated from blood cells and kept frozen until assayed. *PRC* was determined using an immunoradiometric assay from CIS Bio International, Gif-Sur-Yvette Cedex, France. Minimal detection level was 1 pg/ml. The coefficients of variation were 0.9-3.6% (intra-assay) and 3.7-5.0% (inter-assay) in the range of 4–263 pg/ml. *Aldo* was determined by RIA using a kit from Demeditec Diagnostics GmbH, Kiel, Germany. Minimal detection level was 25 pmol/l. The coefficients of variation were 9.0% (inter-assay) and 8.5% (intra-assay). *Ang II* were extracted from plasma with C_18_ Sep-Pak (Water associates, Milford, MA, USA), and subsequently determined by radioimmunoassay [20,21]. The antibody against Ang II was obtained from the Department of Clinical Physiology, Glostrup Hospital, Denmark. Minimal detection level was 2 pmol/L. The coefficients of variation were 12% (inter-assay) and 8% (intra-assay).

### Statistics

Data are presented as means ± standard deviations (±SD), if they showed normality, and as medians with 25% and 75% percentiles in brackets, if normality was not found. Comparisons between groups were performed with unpaired t-test or Mann–Whitney U-test. Paired comparisons within groups were performed with paired t-test or Wilcoxon signed rank test. Pearson’s correlation was used to test for significant correlation between changes in p-BNP and changes in p-25(OH) D. Statistical significance was defined as p < 0.05. Statistical analyses were performed using PASW version 20.0.0 (SPSS Inc.; Chicago, IL, USA).

## Results

### Demographics

**Figure 1:**
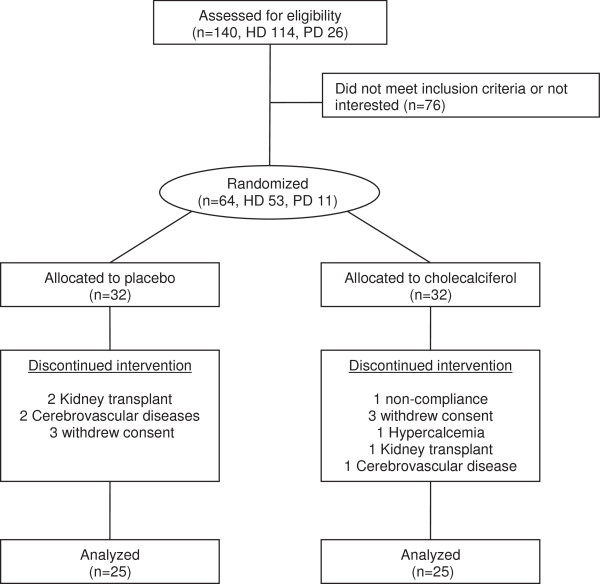
Flow chart.

A total of 140 dialysis patients were screened for participation and 64 patients were included in the trial (Figure 1), and had baseline measurements performed. During the trial 14 patients were with-drawn due to kidney transplantation (3), cerebrovascular disease which made oral ingestion of tablets impossible (3), non-compliance (1), withdrawal of consent (6) or hypercalcemia (1). Thus follow up measurements were performed in 50 patients. Six patients developed hypercalcemia, which was treated within the guidelines of our department. Five of the patients were allocated to the placebo group and one to the cholecalciferol group. The patient in the cholecalciferol group was the patient who was excluded due to persistent hypercalcemia.

Baseline characteristics are shown in Table 1. Baseline p-BNP and p-25(OH) D was similar between groups. At baseline, p-25(OH) D was <80 nmol/L in 47 patients (86%), and <50 nmol/L in 33 patients (66%). Mean compliance by pill count was 99% in both groups.

**Table 1:** Baseline characteristics according to randomization in a randomized, placebo-controlled, double-blinded study of 50 dialysis patients

	**Placebo**	**Vitamin-D3**	**P value**
**Age** (years)	67 ± 13	68 ± 9	0.794
**Body mass index** (kg/m^2^)	23.8 ± 4.4	24.0 ± 4.5	0.856
**Male sex,***n* (%)	15 (60)	17 (68)	0.556
**Smokers,***n* (%)			
Current	5 (20)	1(4)	0.220
Former	5 (20)	6 (24)	
Never	15 (60)	18 (72)	
**Type 1 diabetes,***n* (%)	4 (16)	2 (8)	0.384
**Type 2 diabetes,***n* (%)	5 (20)	2 (8)	0.221
**HD, *n *** **(%)**^**A**^	24 (96)	19 (76)	0.042
Dialysis sessions pr. week for HD patients	3.2 ± 0.6	3.2 ± 0.6	0.817
**Medication,***n* (%)			
ACEi/ARBs	17 (68)	17 (68)	0.914
Calcium channel blockers	7 (28)	13 (54)	0.083
Beta blockers	11 (44)	15 (60)	0.285
Loop diuretics	11 (44)	12 (40)	0.777
Minoxidil	5 (20)	6 (24)	0.733
Alfa-blockers	2 (8)	1 (4)	0.552
Alfacalcidol	12 (48)	15 (60)	0.393
Paricalcitol	1 (4)	1 (4)	1.000
Cinecalcet	2 (8)	2 (8)	1.000
Phosphate binder	17 (68)	20 (80)	0.333
Lanthanum	3 (12)	4 (16)	0.684
Sevelamer	10 (40)	14 (56)	0.258
Calcium-containing^B^	15 (60)	17 (68)	0.254
Erythropoetin analogue	24 (96)	21 (84)	0.157
P-BNP (pmol/L)	81 (24;186)	61 (26;378)	0.600
P-25(OH) D (nmol/L)	28 (20;69)	28 (20;48)	0.614

Values are mean ± SD, *n* with percentage in brackets or medians with 25 and 75 percentiles in brackets. ACEi: Angiotensin converting enzyme inhibitors, ARB: Angiotensin receptor blocker. BNP: Brain natriuretic peptide, 25(OH): 25-hydroxy-vitamin D ^A^One subject changed from peritoneal dialysis to haemodialysis (HD) after two months and was placed in HD. ^B^Unikalk or Osvaren.

### Vasoactive hormones and calcium metabolism

**Figure 2:**
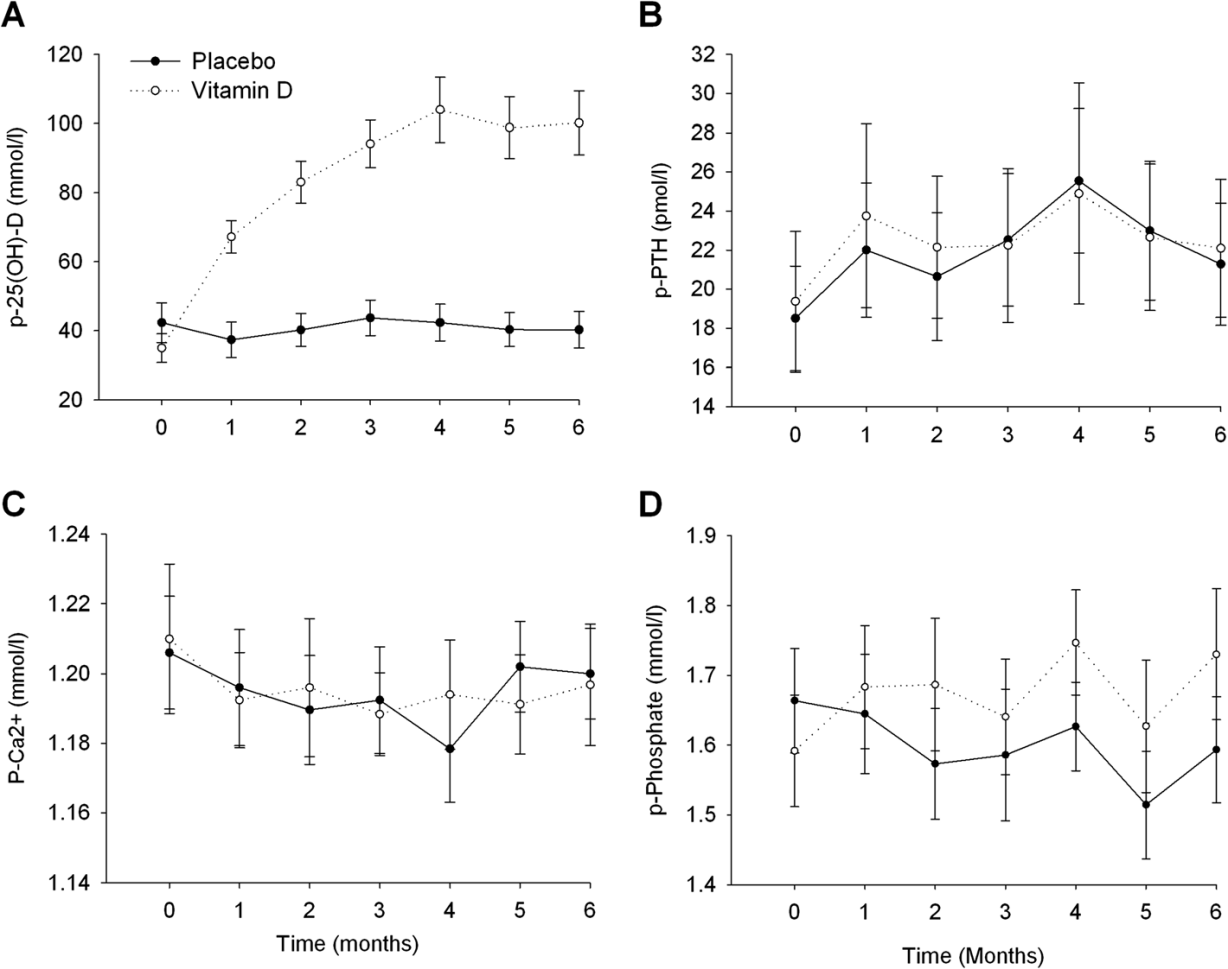
**Mean plasma concentrations of 25-hydroxy-vitamin D (p-25(OH)-D) (A), p-PTH (B), plasma ionised calcium (p-Ca**^**2+**^**) (C) and p-phosphate (D) with SEM (n = 50).**

Changes in p-BNP, PRC, p-Ang II and p-Aldo are given in Table 2. Median changes in p-BNP, p-Ang II, PRC and p-Aldo between baseline and follow-up did not differ at follow-up or were significantly different between groups. Changes in plasma levels of 25(OH) D, PTH, phosphate and Ca^2+^ levels are depicted in Figure 2 with baseline and follow-up values given in Table 2. There was no correlation between changes in p-BNP and changes p-25(OH) D from baseline to follow-up (*r* = -0.069, p = 0.633)

**Table 2:** Effect of cholecalciferol (Vitamin D3) treatment on calcium metabolism and vasoactive hormones in a randomized, placebo-controlled study of 50 patients on dialysis

	**Baseline**	**6 months**	**P value (difference in response)**
**P-25(OH)D (nmol/L)**			
Placebo	28 (20;69)	30 (22;50)	<0.001
Cholecalciferol	28 (20;48)	84 (65;125)*	
**P-PTH (pmol/L)**			
Placebo	18.0 (5.9;30.5)	12.9 (8.7;33.7)	0.986
Cholecalciferol	13.5 (5.8;29.6)	17.4 (9.1;35.3)	
**P-Ca**^**2+ **^**(mg/dL)**			
Placebo	1.20 ± 0.08	1.20 ± 0.07	0.724
Cholecalciferol	1.21 ± 0.11	1.20 ± 0.09	
**P-Phosphate (nmol/L)**			
Placebo	1.66 ± 0.37	1.59 ± 0.38	0.103
Cholecalciferol	1.59 ± 0.40	1.73 ± 0.47	
**P-Alkaline Phosphatase (U/L)**			
Placebo	67 (51;99)	72 (59;93)	0.393
Cholecalciferol	70 (48;92)	73 (48;106)	
**P-CRP (mg/L)**			
Placebo	4.5 (1.7;11.7)	2.5 (1.6;13.9)	0.237
Cholecalciferol	3.4 (1.1;13.3)	3.9 (1.1;11.3)	
**P-leukocytes (10**^**9**^**/L)**			
Placebo	6.3 (5.2;7.7)	6.3 (5.6;7.3)	0.740
Cholecalciferol	6.3 (5.1;7.5)	5.8 (5.2;8.1)	
**P-Haemoglobin (mmol/L)**			
Placebo	7.3 ± 0.8	7,3 ± 0.7	0. 419
Cholecalciferol	7.4 ± 0.8	7.2 ± 0.6	
**P-BNP (pmol/L)**			
Placebo	81 (24;186)	50 (30;265)	0.820
Cholecalciferol	61 (26;378)	95 (35;363)	
**PRC (pg/mL)**			
Placebo	10 (4;19)	8 (6;23)	0.883
Cholecalciferol	6 (4;12)	8 (4;20)	
**P-Ang II (pg/mL)**			
Placebo	12 (7;23)	15 (4;33)	0.167
Cholecalciferol	7 (3;26)	12 (5;60)	
**P-Aldosterone (pmol/L)**			
Placebo	135 (49;300)	209 (68;329)	0.415
Cholecalciferol	125 (61;250)	155 (76;312)	

Values are mean ± SD or medians with 25 and 75 percentiles in brackets. 25(OH): 25-hydroxy-vitamin D, PTH: parathyroid hormone, CRP, C-reactive protein, BNP: Brain natriuretic peptide, PRC: Plasma renin concentration, Ang II: Angiotensin II, P values represent the probability of a difference in response to treatment between groups. Statistical significant difference from baseline: * = p < 0.001.

### 24-h BP and arterial stiffness

No difference in baseline 24-h BP was observed between groups (Table 3). In placebo group 24-h SBP decreased significantly (-9 ± 17 mmHg, p = 0.023) and 24-h DBP in-significantly (-4 ± 10 mmHg, p = 0.068). In cholecalciferol group 24-h SBP and 24-h DBP decreased tended to decrease (-5 ± 24, p = 0.376 and -2 ± 10 mmHg resp., p = 0.396). The reductions in 24-h BP were not different between groups. The same pattern was seen for day-time and night-time values with no difference between groups.

**Table 3:** Effect of 6 months cholecalciferol treatment 24 hour blood pressure (24-h BP) in a randomized, placebo-controlled study of 50 patients on dialysis

	**Baseline**	**6 months**	**P value (difference in response)**
**24-h SBP (mmHg)**			
Placebo	136 ± 22	127 ± 23^†^	0.511
Cholecalciferol	135 ± 18	130 ± 14	
**24-h DBP (mmHg)**			
Placebo	73 ± 10	69 ± 10	0.451
Cholecalciferol	73 ± 9	71 ± 8	
**24-h heart rate (bpm)**			
Placeb	74 ± 8	74 ± 7	0.464
Cholecalciferol	70 ± 9	71 ± 8	
**24-h daytime SBP (mmHg)**			
Placebo	138 ± 22	130 ± 22^†^	0.504
Cholecalciferol	137 ± 18	133 ± 15	
**24-h daytime DBP (mmHg)**			
Placebo	75 ± 11	71 ± 11	0.519
Cholecalciferol	74 ± 9	72 ± 8	
**24-h daytime heart rate (bpm)**			
Placebo	75 ± 8	76 ± 8	0.586
Cholecalciferol	71 ± 9	73 ± 9	
**24-h nighttime SBP (mmHg)**			
Placebo	131 ± 26	121 ± 27^†^	0.498
Cholecalciferol	129 ± 19	125 ± 17	
**24-h nighttime DBP (mmHg)**			
Placebo	69 ± 11	65 ± 11^†^	0.361
Cholecalciferol	69 ± 9	68 ± 11	
**24-h nighttime heart rate (bpm)**			
Placebo	70 ± 8	70 ± ± 6	0.270
Cholecalciferol	67 ± 9	69 ± ± 9	

Values are means ± SD. P values represent the probability of a difference in response to treatment between groups. Statistics are performed with t-test. Statistical significant difference from baseline compared with paired t-test. Statistical significant difference from baseline: ^†^ = p < 0.05.

cBP and measures of arterial stiffness are documented in Table 4. Data on PWA and PWV were available on 41 and 40 patients, resp. No change between and within groups was found in cBP and PWV. AIx decreased significantly in placebo group and was unchanged in cholecalciferol group, and the response was significantly different between groups.

**Table 4:** Effect of 6 months cholecalciferol treatment on pulse wave velocity (PWV), augmentation index (AIx), and central diastolic and systolic blood pressure (cDBP, cSBP) in a randomized, placebo-controlled study of 50 patients on dialysis

	**Baseline**	**6 months**	**P value (difference in response)**
**PWV (m/s)**			
Placebo (n = 19)	10.0 ± 2.0	10.1 ± 2.5	0.269
Cholecalciferol (n = 22)	9.7 ± 2.5	10.5 ± 4.0	
**AIx (%)**			
Placebo (n = 22)	26.4 ± 11.5	22.2 ± 11.2^†^	0.013
Cholecalciferol (n = 18)	22.1 ± 9.7	24.6 ± 12.7	
**cSBP (mmHg)**			
Placebo (n = 22)	127 ± 24	126 ± 26	0.744
Cholecalciferol (n = 18)	128 ± 14	125 ± 17	
**cDBP (mmHg)**			
Placebo (n = 22)	68 ± 11	70 ± 11	0.779
Cholecalciferol (n = 18)	71 ± 9	71 ± 12	

Values are means ± SD or medians with 25 and 75 percentiles in brackets. Statistics are performed with t-test. P values represent the probability of a difference in response to treatment between groups: Significant difference from baseline: ^†^ = p < 0.05.

### Echocardiography

Transthoracic echocardiography measures at baseline and 6 months follow-up are shown in Table 5.

**Table 5:** Effect of six months cholecalciferol treatment on cardiac structure and function measured by transthoracic echocardiography in a randomized, placebo-controlled study of patients on dialysis

	**Baseline**	**6 months**	**P value (difference in response)**
**Left ventricular end-diastolic volume (biplane), ml**			
Placebo (n = 24)	109 ± 38	101 ± 43	0.024
Cholecalciferol (n = 22)	99 ± 33	108 ± 42	
**Left ventricular ejection fraction (biplane), %**			
Placebo	52 ± 14	52 ± 17	0.515
Cholecalciferol	53 ± 14	56 ± 12	
**Left ventricular mass index, g/m2**			
Placebo	116 ± 36	111 ± 39	0.397
Cholecalciferol	123 ± 34	127 ± 50	
**Left atrial volume index, ml/m2**			
Placebo	35 ± 14	37 ± 14	0.637
Cholecalciferol	40 ± 16	41 ± 14	
**Early/late mitral inflow wave velocity ratio (E/A)**			
Placebo	0.84 ± 0.21	0.89 ± 0.28	0.501
Cholecalciferol	1.10 ± 0.59	1.21 ± 0.72	
**Transmitral E-wave deceleration time, s**			
Placebo	228 ± 92	230 ± 76	0.455
Cholecalciferol	226 ± 62	215 ± 61	
**Early mitral inflow wave velocity/early diastolic mitral annular velocity ratio (E/e’)**			
Placebo	12 ± 6	13 ± 5	0.078
Cholecalciferol	15 ± 8	17 ± 9	

Values are means ± SD. P values represent the probability of a difference in response to treatment between groups. Data was available for 24 patients in cholecalciferol group and 22 patients in the placebo group. Statistics are performed with t-test. Statistical significant difference from baseline compared with paired t-test.

LV end diastolic volume increased significantly in the cholecalciferol group compared to placebo. Cholecalciferol treatment had no effect on LVEF, LVMI, LAVI, E wave deceleration time, E/A ratio or E/e’. Furthermore cholecalciferol treatment had no impact on mitral plane systolic motion, global or regional strain (data not shown).

### Medication

Changes in calcium supplements, alfacalcidol, paricalcitol, calcimimitecs, phosphate binders and erythropoietin analogues from baseline to follow-up are shown in Table 6. Median changes were 0 for all the drugs in Table 6, indicating that the dose prescribed at inclusion was continued throughout the study period in a majority of the patients. There was no difference in mean dose change for any of the drugs between groups.

**Table 6:** Changes in calcium supplements, alfacalcidol, paricalcitol, calcimimitecs, phosphate binders and erythropoietin analogues in a randomized, placebo-controlled study investigating the effects of cholecalciferol supplementation for 6 months in 50 patients on dialysis

	**Vitamin D analogues**		**Calcimimetic**	**Phospahte binders**				**Erythropoietin analogues**	
	**Alfacalcidol**	**Paricalcitol**	**Cinecalcet**	**Unikalk**^**A**^	**Sevelamer**	**Lanthanum**	**Osvaren**^**B**^	**Epoetin**	**Darbapeotin**
**Placebo (changes from baseline)**									
Increased dose (*n*)	7	0	1	0	6	1	7	1	7
Same dose (*n*)	10	25	23	22	13	23	12	23	6
Reduced dose (*n*)	8	0	1	3	6	1	6	1	12
Mean change in dose	0.2 ± 4.3 μg/week	0 ± 0 μg/week	0.0 ± 26 mg/day	-0.4 ± 1.0 tablets/day	0.0 ± 2.4 g/day	30 ± 340 mg/day	-0.1 ± 2.8 tablets/day	160 ± 2939 IU/week	-4.8 ± 22.6 μg/week
**Cholecalciferol (changes from baseline)**									
Increased dose (*n*)	8	1	1	2	3	3	3	0	9
Same dose (*n*)	12	23	22	17	14	18	17	25	6
Reduced dose (*n*)	5	1	2	6	8	4	5	0	10
Mean change in dose	0.7 ± 3.0 μg/week	0 ± 4 μg/week	-2.4 ± 21 mg/day	-0.6 ± 1.4 tablets/day	-0.6 ± 1.8 g/day	30 ± 931 mg/day	-0.4 ± 2.0 tablets/day	0 ± 0 IU/week	3.2 ± 34.0 μg/week

Mean change in dose is shown as mean ± SD ^A^One tablet contains 1000 mg calcium acetate ^B^One tablet contains 435 mg calcium acetate and 235 mg magnesium carbonate.

The Number of patients, who had changes in antihypertensive medication, is shown in Table 7. Median changes were 0 for every antihypertensive drug. In placebo group, 33 dose-increases and 16 had dose-reductions were observed, and in cholecalciferol group and in cholecalciferol group 29 dose-increases and 23 dose-reductions were observed.

**Table 7:** Changes in antihypertensive mediation in a randomized, placebo-controlled study investigating the effects of cholecalciferol supplementation for 6 months in 50 patients on dialysis

**Changes from baseline**	**ACE-inhibitor**	**AT2-antagonist**	**Calcium-antagonist**	**Beta-blocker**^**A**^	**Fourosemide**	**Minoxidil**	**Alfa-blocker**	**Moxonidine**
**Placebo**								
Increased dose (*n*)	6	4	4	9	6	4	0	0
Same dose (*n*)	18	19	19	14	15	16	25	25
Reduced dose (*n*)	1	2	2	2	4	5	0	0
**Cholecalciferol**								
Increased dose (*n*)	3	5	3	4	7	4	2	1
Same dose (*n*)	16	19	18	18	14	18	23	22
Reduced dose (*n*)	6	1	4	3	4	3	0	2

ACE: Angiotensin converting enzyme, AT2: Angiotensin 2. ^A^1 patient in placebo and 1 patient in cholecalciferol group had metroprolol substituted with carvedilol in equivalent doses and is reported as given same dose beta-blocker.

## Discussion

This is the first randomized, placebo-controlled, double-blinded, single-centre intervention study investigating the effects of cholecalciferol supplementation on cardiac function in dialysis patients. Previous intervention studies, which found indication that cholecalciferol treatment may reduce LVMI and BNP in patients on chronic dialysis, were prospective uncontrolled studies [3,17]. We hypothesized that daily cholecalciferol supplementation in dialysis patients improves cardiac function and reduces 24-h BP and arterial stiffness. Cholecalciferol caused a marked increase in p-25(OH) D without increased incidence of hypercalcemia or other adverse effects. After six months, no significant differences were observed in p-BNP, cardiac function, 24-h BP or arterial stiffness between groups.

Previously, intra-venous 1,25(OH)_2_D (calcitriol) reduced LV hypertrophy in patients on hemodialysis with elevated p-PTH [22,23]. Two prospective studies have demonstrated a reduction in LVMI and p-BNP after 6 months of cholecalciferol supplementation [3,17], which is in contrast to our findings where no change was found. In this study 3,000 IU were given daily. Buchales and colleagues administered a higher (x 1.5) dose and Mathias and colleagues used a substantial higher dose (x 2.4) in HD patients with p-25(OH) D < 37 nmol/L but a lower dose (x 0.4 – 0.5) in patients with p-25(OH) D > 37 nmol/L, than we used in this study [3,17]. As in the previous studies, we found an increased in p-25(OH) D levels in response to cholecalciferol supplementation. Dialysis patients have reduced renal mass and reduced renal 1α-hydroxylase activity [24], and although cholecalciferol supplementation increased p-1,25(OH)_2_D in patients on dialysis, the increase is impaired compared to non-dialysis populations [1,3,25,26]. Vitamin D receptors and 1α-hydroxylase is present in cardiac tissue [9,27], but the increase in p-1,25(OH) D may not be sufficient to activate the vitamin D receptor in cardiac tissue, with the doses used in this study. However, antihypertensive medication was changed during the study, which could conceal the effects of cholecalciferol.

We found an increase in LV end-diastolic volume after 6 months of cholecalciferol treatment compared to placebo. This is not a beneficial effect, but the difference is small and there were no differences with-in groups from baseline to follow-up in LV end-diastolic volume. This could be by chance and further studies will have to clarify this.

Studies evaluating the effect of cholecalciferol on BP have shown mixed results [28-30]. Two recent trials in hypertensive patients measured BP as 24-h BP, and could not demonstrate reductions in BP by cholecalciferol supplementation [31,32], but a sub-group analysis of 25(OH) D deficient patients showed a small but significant reduction in 24-h BP [31]. We found a tendency towards a reduction of 24-h BP in cholecalciferol group, but surprisingly an even larger and significant reduction in BP in the placebo group. Blood pressure medication was recorded at baseline and follow-up. In the placebo group, 33 dose-increases and 16 dose-reductions (difference of 17) were recorded and in cholecalciferol group 29 dose-increases and 23 dose-reductions (difference of 6) were observed. There seems to be a majority of dose-increases in the placebo group, which might explain the reduction in BP seen in placebo group. In hemodialysis patients BP is shown to increase in the inter-dialysis period [33]. Since BP is dependent on volume status, an improvement of volemic state might explain the reduction in BP. Hence, changes in 24-h BP are likely related to changes in medication and volume status. Our results do not suggest a benefit of cholecalciferol supplementation on 24-BP, but BP reductions may induced by cholecalciferol may be concealed by changes in volume status and medication. Thus, we can not exclude that cholecalciferol might have beneficial effects on BP.

PWV is a predictor for all-cause and cardiovascular mortality in patients with end-stage renal disease [34] and we found no change in PWV in the groups at follow-up, which is in agreement with previous reports [1,35]. PWV tends to increase in the inter-dialysis period [36], most likely due to volume expansion. Hemodialysis patients had PWV measurements performed just prior to a dialysis session where volume-expansion is at its highest and this could conceal effects of cholecalciferol treatment. AIx decreased significantly in placebo group and was unchanged in cholecalciferol group. Most likely this is explained by the lower 24-h BP in the placebo group. Previously cholecalciferol supplementation did not change AIx [1], and this needs further clarification.

Vitamin D may play an important role in the modulation of renin-angiotensin system in patients on dialysis. In vitamin D receptor knock-out mice renin expression is highly increased leading to hypertension and LV hypertrophy [37] and in mice active vitamin D analogue treatment suppresses renin expression, independent of PTH [38]. In dialysis patients, high dose intravenous vitamin D analogue treatment led to a significant reduction in PRC and p-Ang II [23]. In contrast, PRC and p-Ang II was unchanged after active vitamin D analogue supplementation in patients with CKD stage III-IV [39] and type 2 diabetics with nephropathy [40] and after 6 months oral cholecalciferol supplementation in hypertensives [31]. This is in agreement with the finding in the present trial. Although we recorded no effect of cholecalciferol on circulating concentrations, these measurements might not be accurate indicators of renin or angiotensin II activity within the kidney in response to cholecalciferol. In addition, given that patients were non-fasting and 68% were on angiotensin II receptor blocker or angiotensin-converting enzyme inhibitor, conclusions regarding changes in the RAS should be made with caution.

Previous conclusions from clinical trials regarding the effect of vitamin D supplementation on PTH in dialysis patients are inconsistent, with both suppressed [3,41,42] and unchanged PTH [1,35,43-45] observed. In this study, cholecalciferol plasma concentrations of calcium, phosphate and PTH were unchanged. However, in this trial setting the effect of cholecalciferol treatment is very difficult to estimate. Cholecalciferol was used on top of usual treatment and changes in p-calcium, p-phosphate and PTH during the trial were regulated tightly with-in the usual guidelines of our department. Although medication changes regarding calcium and phosphate metabolism in the 6 month treatment period were small and did not seem different between groups, potential changes in plasma concentrations of phosphate, calcium and PTH are most likely concealed by medication changes.

Oral supplementation of 3,000 IU cholecalciferol daily was highly effective in restoring p-25(OH) D levels without increasing the risk of hypercalcemia or other adverse effects. The incidence of persistent hypercalcemia, was after the trial was finished associated with malignant disease, and was most likely not related to cholecalciferol supplementation.

The design as a randomised, placebo-controlled, double-blinded, single-centre trial was an essential strength of the present study. Compliance to study medication, documented by pill count, was high and a relatively high cholecalciferol was given.

Usual dialysis and medical treatment, except nutritional vitamin D supplementation, was continued during study period, and adjusted as clinically warranted during the course of the trial. We did not find it ethically justified to discontinue usual medication or with-hold optimal treatment. Thus, the study was performed under optimal care, and this may influence effect variables and conceal the effects of cholecalciferol treatment. Patients were included regardless of their p-25(OH) D levels. Thus, some patients were had sufficient vitamin D levels. In patients with sufficient vitamin D levels, raising vitamin D levels further may not change outcomes, and this could conceal potential beneficial effects of cholecalciferol supplementation in vitamin deficient patients. Further investigations in vitamin D deficient dialysis patients are needed the address this problem.

We have not performed sub-group analysis since this study was not powered for such analysis. For instance, both patients on HD and PD were included in this study, but the study was not powered to reveal a difference in response to cholecalciferol between the different dialysis modalities. Hence, it cannot be excluded that certain sub-groups might benefit from cholecalciferol supplementation.

This study was powered to detect a difference in p-BNP. For some of the secondary effect variables this study may not have sufficient power to detect a difference. We cannot exclude that a bigger sample size could reveal differences in some of the secondary effect variables.

## Conclusions

In conclusion, six months of cholecalciferol treatment in patients on chronic dialysis did not improve 24-h BP, arterial stiffness or cardiac function. The study population was a heterogeneous group of patients on dialysis on usual medication, and although we did not find beneficial effects of cholecalciferol supplementation, certain subgroups could benefit from cholecalciferol supplementation. Further studies are warranted to evaluate this.

## Competing interests

All authors declare no conflict of interests. Ferrosan A/S supplied cholecalciferol and placebo free of charge, but did not play any role in the study.

## Authors’ contributions

FHM, TL, JNB and EBP designed the project. BJ, RK and HV performed echocardiography, TL, FHM. IMT, AEO, JNB, and EBP recruited and had daily contact to patients. ABH were responsible for measurements of p-25(OH), p-PTH, p-BNP and routine laboratory analyses. EBP was responsible for measurements of PRC, p-Ang II and p-Aldo. FHM and TL were responsible for applanation tonometry measurements. FHM, TL, ASPK, IMT, AEO, JNB and EBP were responsible for 24-h BP measurements. FHM wrote the primary draft. The final manuscript was prepared in collaboration between FHM, HV, TL, JNB and EBP. All authors read and approved the final manuscript.

## Pre-publication history

The pre-publication history for this paper can be accessed here:

http://www.biomedcentral.com/1471-2369/15/50/prepub
